# Small target detection in coal mine underground based on improved RTDETR algorithm

**DOI:** 10.1038/s41598-025-96638-8

**Published:** 2025-04-08

**Authors:** Feng Tian, Cong Song, Xiaopei Liu

**Affiliations:** 1 College of Communication and Information Technology, Xi’an University of Science and Technology, Xi’an, 710054 China; 2 Xi’an Key Laboratory of Network Convergence Communication, Xi’an, 710054 China

**Keywords:** Underground coal mines, Small target detection, Attention mechanism, RTDETR, Feature extraction, Electrical and electronic engineering, Computer science

## Abstract

Aiming at the problem of low detection accuracy of small targets such as helmets and self-rescuers in complex scenarios in coal mines, a small target detection method based on improved Real-Time DEtection TRansformer (RTDETR) for underground coal mines is proposed. A new BasicBlock-PConv module was created by incorporating Partial Convolutions (PConv) into the conventional BasicBlock, which was based on the FasterNet network. This decreased the number of network parameters and computation. By introducing Deformable Attention in the coding part of the RTDETR algorithm, the deformable feature of this attention mechanism is used to improve the network’s ability to extract effective image features. In order to increase the accuracy of tiny object detection and concentrate on the detail information in the shallow feature map, the small object detection layer P2 is simultaneously added to the Head of the coding section. Based on the improvement of the above three parts, the improved PDP-RTDETR working model in this paper achieves a Mean Average Precision (mAP) of 56.6% for detecting small targets on the self-constructed dataset, which is 11.2, 12.1, 9.9, and 5.2% better than that of the traditional models Yolov5s, Yolov7-Tiny, Yolov8n, and RTDETR, respectively. Meanwhile, the improved PDP-RTDETR algorithm parameter count is reduced by 2.6 M compared to the base model. The results suggest that the approach can successfully increase the detection accuracy of small targets in the mine scene, which gives a certain reference value for the application of small target detection.

## Introduction

With the increase in demand for coal in the iron and steel, chemical and other industries, the consumption of coal has been promoted. However, coal mining is still a high-risk operation, with a complex and changeable underground environment and a large number of operators, which is very prone to all kinds of safety hazards and thus causes safety accidents. Video surveillance is regarded as a crucial tool for monitoring coal mine safety, as it allows any risky behavior or irregularities in the coal miners’ operations to be quickly identified. One of the more common unsafe behaviors is the incorrect wearing and use of safety equipment such as helmets and self-rescuers. If targets such as helmets and self-rescuers can be accurately detected, it is significant for the safety of mine workers. Low detection accuracy is a problem because the operating environment of underground coal mines is extremely complex, the monitoring camera is fixed in place, has wide coverage, and is far away from the subject. Additionally, small protective equipment^1^, like safety helmets and self-rescuers, is easily affected by changes in the target’s size and the underground environment during the recognition process.

Currently, the vast majority of coal mining companies rely on video surveillance systems to assist in production safety management and underground production scheduling. However, the supervision of violation behaviours of underground operators mainly relies on real-time video monitoring and historical video playback. Although these two methods have expanded the coverage of safety supervision to a certain extent, manual supervision still faces the following two major problems: firstly, the energy of safety supervisors is limited, the underground operating environment is complex and the monitoring screen is huge, so with the extension of working time, the personnel are prone to visual fatigue, which makes them unable to effectively supervise each frame, thus missing the opportunity of timely detection and stopping unsafe behaviour; secondly, the opportunity of viewing the video surveillance system is limited, which makes them unable to effectively supervise each frame, thus missing the opportunity of timely detection and stopping unsafe behaviour. Secondly, although viewing historical video data can trace and deal with unsafe behaviours that have occurred, due to the lag in data processing, it is not possible to eliminate potential safety hazards that have occurred in a timely manner, and it is difficult to achieve effective prevention of potential accidents and real-time response.

In recent years, the rapid development of deep learning technology has greatly promoted the improvement and optimisation of target detection algorithms, especially in improving detection accuracy and speeding up the detection speed has made significant progress. However, in actual scenarios, the target detection effect is still affected by factors such as background environment changes, equipment occlusion, and scale changes, which may lead to misdetection and omission of the detection system, in addition, in coal mine scenarios, safety helmets and self-rescuers are more difficult to detect as relatively small targets. Given this, the main focus of this article is the issue of recognizing small targets in underground coal mine sceneries.The following are the papers primary contributions:

- Using a partially convolved PConv in the Backbone network, which is based on the RTDETR model, the network can efficiently manage inputs with irregular or missing data, resulting in a lightweight model that maintains accuracy on a range of visual tasks.
- By incorporating a visual Transformer mechanism with deformable attention in the AIFI’s encoding section, the model is able to more flexibly capture local features and dynamically modify the attention’s position based on the input image’s content.
- Lastly, by including a small target detection layer P2 in EncoderHead, more positional information and specific features about small targets are preserved to increase target detection accuracy.
- Following extensive testing in comparison to other popular target detection methods, PDP-RTDETR has significantly improved in various performances, proving its effectiveness.

## Related work

Traditional detection algorithms tend to have insufficient expressive ability and poor feature separability for the target, as well as the problems of slow detection speed and high computational cost. In order to solve the limitations of traditional methods, target detection algorithms based on deep learning are widely used, such as Faster R-CNN^2^, You Only Look Once (YOLO)^3^, RTDETR and so on. The powerful feature learning ability of deep learning makes it have higher detection speed and accuracy, which can better complete the task of detecting small targets in the mine scene.

To tackle the difficulties of extracting effective feature information for small target detection and achieving low detection accuracy in complex scenes, Chen et al.^4^ introduced the PConv architecture in the C2 module in order to optimise the increase in parameters and FLOPs brought about by the use of the YOLOv8 module and used the C2f-PConv module instead of the multiple convolution operation of the CSP module, thus reducing feature redundancy. Wu et al.^5^ propose a high-precision PC-EMA module based on YOLOv8 in order to solve the problems of low accuracy of traditional methods for detecting surface defects of wind turbines and the difficulty of lightweight deployment, which combines partial convolution with a multi-scale attention mechanism, replacing the bottleneck layer of the YOLOv8 backbone network, thus improving the target feature extraction capability. Su et al.^6^ introduced a scope-C2f module and a perceptually enhanced convolution module based on the YOLOv8 network model. These modules increase the network’s capacity for feature extraction while also improving its ability to capture the finer details of small targets, thereby increasing the accuracy of aerial target detection. Qu et al.^7^ proposed a small object detection algorithm based on an improved YOLOv5s framework to address the issue of low detection accuracy in small traffic sign recognition models under varying weather conditions. By incorporating an additional prediction head into the YOLOv5s network, this approach effectively extracts fine-grained features of small targets from the shallower layers, significantly reducing the missed detection rate of small traffic signs. Huang et al.^8^ proposed an innovative small target detection method based on YOLOv4 to address the issue of small defects generated during the manufacturing processes in the semiconductor and electronics industries. Their approach enhances small target detection efficiency by incorporating shallow feature fusion and optimizing the number and size of anchor frames through k-means++ clustering. The results demonstrate significant improvements in detection performance, highlighting the method’s effectiveness for industrial applications. Qu et al.^9^ proposed a new neural network model, YOLOv8-LA, to develop an AP-FasterNet architecture for small targets commonly found in underwater datasets, with selective processing of the input channels to optimize spatial feature extraction, which allows the model to maintain high detection accuracy while maintaining real-time processing capabilities. Lin et al.^10^ proposed an enhanced feature extraction network based on deformable convolution based on the YOLO7 architecture for the problem that targets in remote sensing images are difficult to detect because they are small and tightly arranged, and this method effectively improves the detection accuracy of small targets in remote sensing images. Jin et al.^11^ used actual imaging condition parameters to decompose image features into domain-invariant and domain-specific features, and the composite features can improve the domain generalisation ability and the accuracy of individual domains compared to the traditional fine-grained domain detection methods, and improve the problem of feature information loss due to the multiscale problem. MITTAL et al.^12^ proposed a cascaded FPN-Cascade architecture based on R-CNN by increasing the threshold value. This approach incorporates an efficient feature pyramid network to address scale variations in target detection by combining information from multiple feature layers, enabling the detection of smaller targets. Chen et al.^13^ multiscale task designed detection framework MSP-YOLO, which introduces an attention-based feature fusion module injected with dilated convolutional clusters, while proposing a multiscale-aware YOLO with super-resolution reconstruction branching, which improves the sensitivity of small object detection. SHANG et al.^14^ enhanced the YOLOv5 algorithm by introducing a non-parametric attention M-SimAM module in the backbone network to improve feature extraction. They added a small target detection layer in the neck network and implemented an enhanced bi-directional feature pyramid (Mul-BiFPN) structure to increase detection speed and accuracy. Additionally, they utilized Focal EIoU as the localization loss function to improve target regression accuracy. Qiu et al.^15^ proposed a pixel-level local contrast measurement method to address the low detection rate associated with small targets having irregular dimensions or complex backgrounds. They designed a multi-scale sliding window for rapid extraction of candidate target pixels and employed a local window based on random wandering for pixel-level segmentation, resulting in a higher detection rate compared to the baseline algorithm. Xu et al.^16^ developed a small target detection algorithm for UAV images based on the enhanced YOLOv8 model, YOLOv8-MPEB. They designed a small target detection layer and integrated an efficient multiscale attention mechanism into the convolutional features to optimize multiscale feature extraction and enhance the accuracy of small target detection in aerial images.

Although the existing methods have improved the small target detection effect to a certain extent, they are mostly for conventional scenes. Due to the complex environment of coal mine underground, there are problems such as insufficient light, serious dust interference, and complex background; it is difficult to extract effective target feature information in the actual detection process, which makes the detection accuracy lower.To address these issues, this paper proposes a lightweight, real-time and efficient PDP-RTDETR small target detection algorithm for coal mine scenes to better protect the safety of mine workers.

## RTDETR network model

**Fig. 1:**
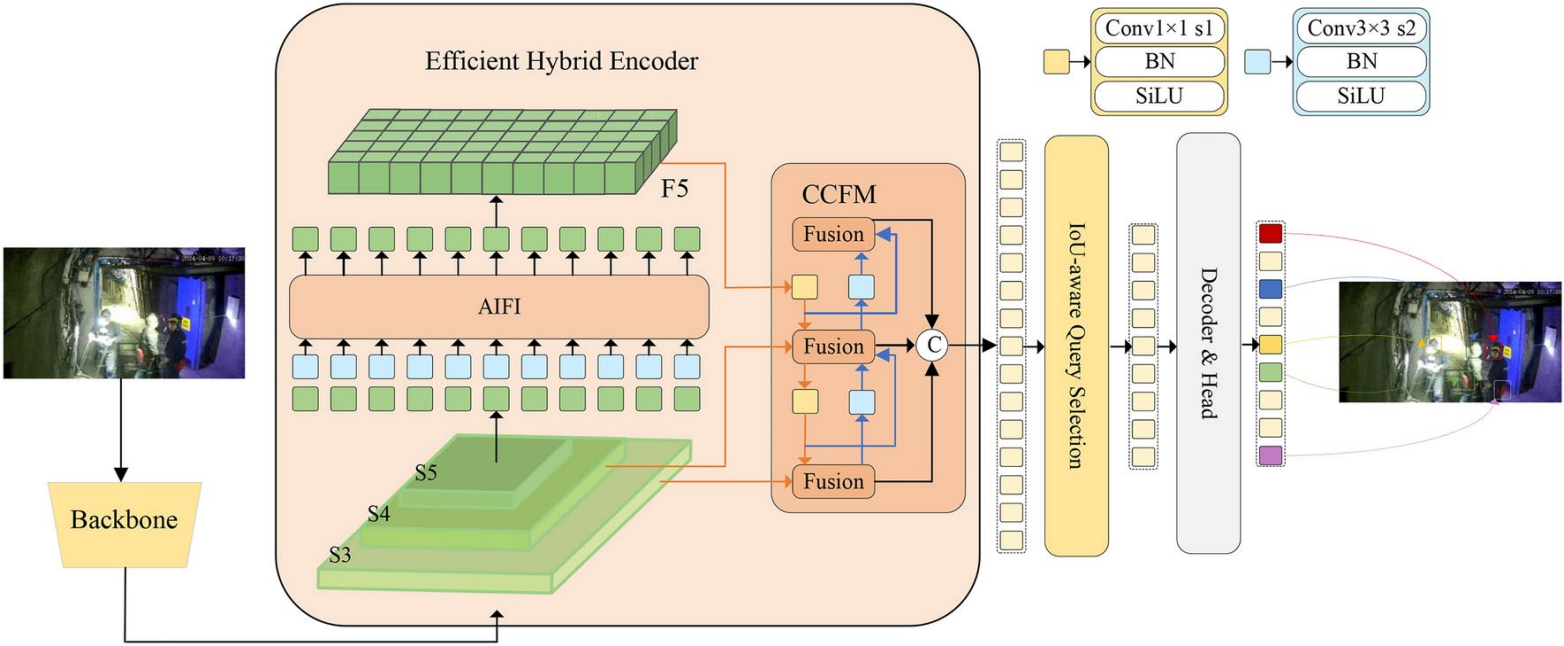
RTDETR network architecture.

RTDETR^17^ is a practical end-to-end real-time object detection model that eliminates anchor-based preprocessing and Non-Maximum Suppression (NMS) post-processing. Compared to other object detection networks, it achieves real-time performance with reduced computational load while maintaining accuracy. Three essential parts comprise the RT-DETR network model: the Transformer Decoder, the Efficient Hybrid Encoder, and the Backbone, which includes an auxiliary prediction header. Hybrid encoders convert multi-scale features into image feature sequences through internal scale interaction and cross-scale fusion. An IoU-aware query selection mechanism is employed to select a fixed number of image features from the encoder’s output sequence as initial object queries for the decoder. The decoder, along with the auxiliary prediction header, iteratively optimizes the object queries to generate bounding boxes and confidence scores. The new structural design enabled RT-DETR to achieve superior results in comparison experiments on the COCOval2017 dataset, outperforming all YOLO^18^ detectors of similar size in both speed and accuracy. The backbone network of the RTDETR model is composed of a residual network, and the residual structure can be classified into two types: one is BasicBlock, which is mainly applied to the ResNet-18 and ResNet-34 models, and the other is BottleNeck, which is mainly applied to the ResNet-50, ResNet-101, and ResNet-152 models. In this paper, considering the complex mine environment, in order to ensure the training speed, the ResNet-18 network with fewer layers is used as the base network, and the RTDETR network structure is shown in Fig. 1.

## PDP-RTDETR small target detection method

**Fig. 2:**
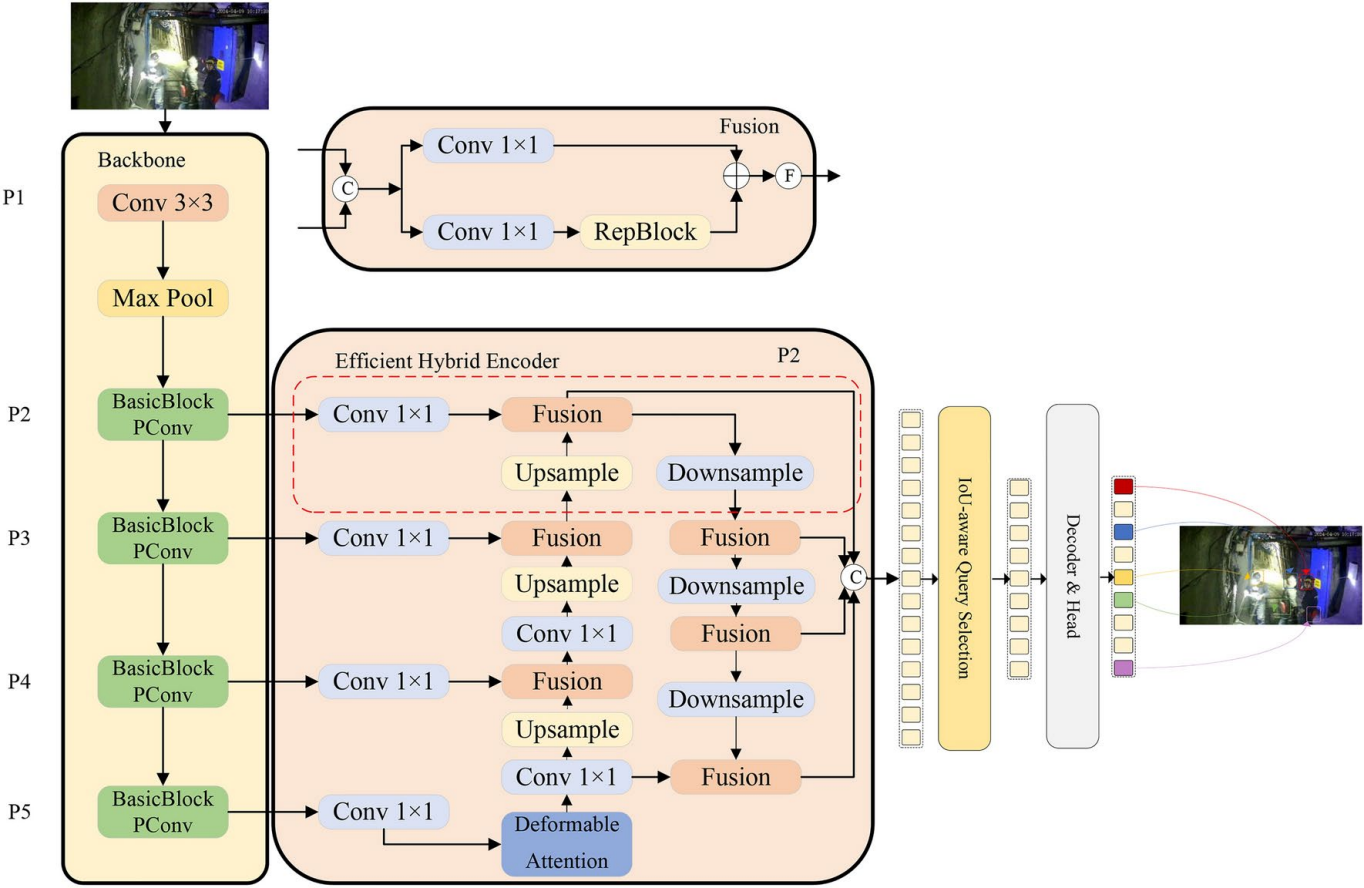
PDP-RTDETR network structure.

Aiming at the problem of low accuracy of small target detection under the mine, a model PDP-RTDETR focusing on small target detection under the mine is proposed according to the characteristics of small targets, and the network structure of the improved algorithm is shown in Fig. 2. Firstly, a lightweight feature extraction module BasicBlock-PConv is used to replace the BasicBlock module in the original network in order to solve the network computational redundancy and accelerate the network computational process; a deformable attention mechanism is introduced in the RTDETR coding part, which is flexible enough to allow the self-attention module to focus on the relevant region and capture the small target information in the high-resolution image. Finally, the small target detection layer P2 is added to the encoder to enhance the network’s extraction of shallow downhole small target features, improve target detection accuracy, and reduce the leakage and false detection rates.

### Improvements to the BasicBlock module

**Fig. 3:**
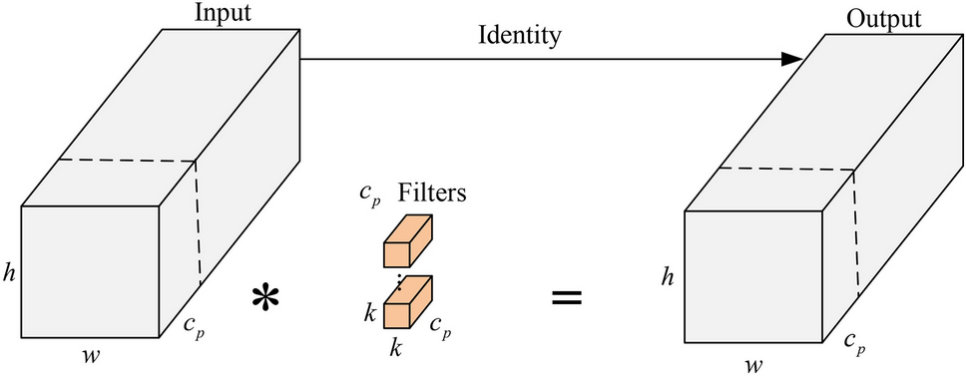
PConv module.

Real-time performance in mines is highly demanded, and under resource limits, lightweight design may guarantee effective data processing and target identification, thereby guaranteeing the real-time performance of underground monitoring systems. The RTDETR feature extraction module (BasicBlock) exhibits significant redundant computations, leading to increased overall computation and model latency. We enhance BasicBlock by incorporating PConv^19^ module from FasterNet. This integration effectively reduces FLOPs, improving detection speed while increasing FLOPS. As a result, the overall computation volume is decreased, enhancing processing speed without compromising accuracy. The structure is illustrated in Fig. 3.

Partial convolution utilizes the redundancy in feature maps by applying regular convolution for spatial feature extraction on specific input channels while ensuring that the remaining channels remain unchanged. For consecutive or regular memory accesses,it computes the first or last consecutive $c_p$ channel as a representation of the entire feature map. The input and output feature maps are typically assumed to have the same number of channels. The FLOPs for PConv are calculated as follows:

1 $$\begin{aligned} \displaystyle O = h \times w \times c_p^2 \times k^2 \end{aligned}$$

where *w* and *h* denote the width and height of the convolution kernel, respectively, and *k* represents the size of the convolution kernel. PConv can reduce the number of floating point operations FLOPs to: as compared to the conventional method of doing convolution operations (*Conv*) on all channels (*c*) of the feature map.

2 $$\begin{aligned} \displaystyle r_F&= \frac{{h \times w \times {k^2} \times c_p^2}}{{h \times w \times {k^2} \times {c^2}}} \nonumber \\ \displaystyle&= \frac{{h \times w \times {k^2} \times \left( \frac{c}{r} \right) ^2}}{{h \times w \times {k^2} \times {c^2}}} \nonumber \\ \displaystyle&= \frac{1}{{r^2}} \end{aligned}$$

Let ${r_F}$ represent the ratio of the number of floating-point operations for PConv to that of conventional convolution, and *r* denotes the channel reduction factor. If $r = {c_p}/c = 1/4$ is the ratio of the PConv part of the channels, then the computation is reduced to that of conventional convolution1/16.

**Fig. 4:**
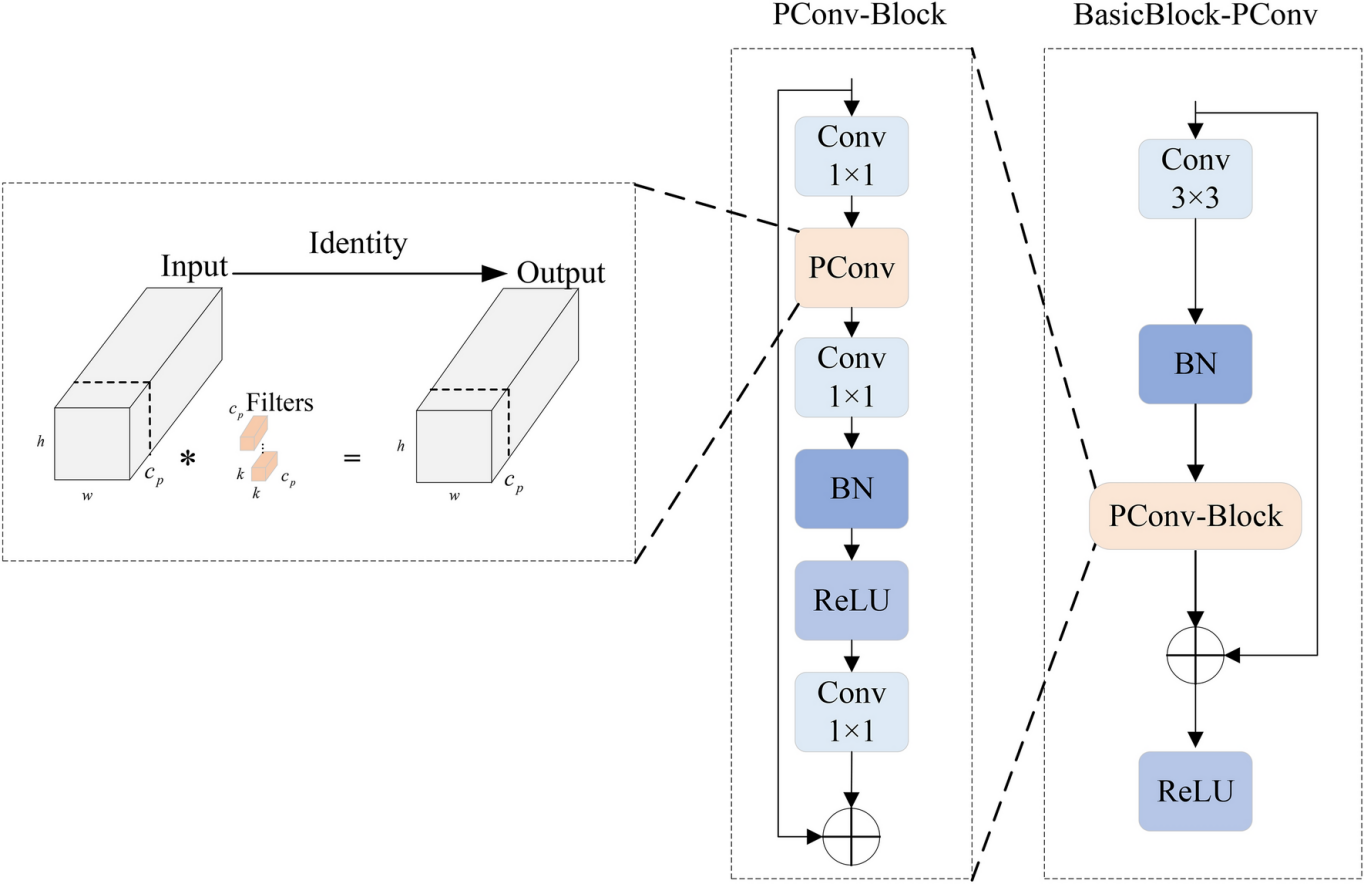
BasicBlock-PConv network module.

The advantage of PConv (Partial Convolution) lies in its selective convolution approach, where it operates only on a subset of input channels instead of convolving all channels. This significantly decreases the computational load of the network and optimizes the model’s performance. The BasicBlock-PConv module enables a high-efficiency and lightweight detection model for downhole small targets. The improved structure is illustrated in Fig. 4.

### Deformable attention mechanism

The image quality is impacted by various scenes, operational settings, and camera angles during the gathering of mining datasets. As a result, the photos exhibit notable variations in backdrop intricacy, scale, and other factors. Furthermore, these variations make further data processing and analysis more difficult. The Self-Attention mechanism in the AIFI module of the RTDETR model primarily focuses on high-level feature map extraction, resulting in the loss of some low-level features when detecting small targets. This loss diminishes its information capture capability. To address the feature loss associated with multi-scale changes and the attention mechanism, the deformable attention^20^ is introduced. The deformable attention mechanism dynamically adjusts weights in feature mapping by focusing on the significance of each region, thereby enhancing the capture of small target features.

**Fig. 5:**
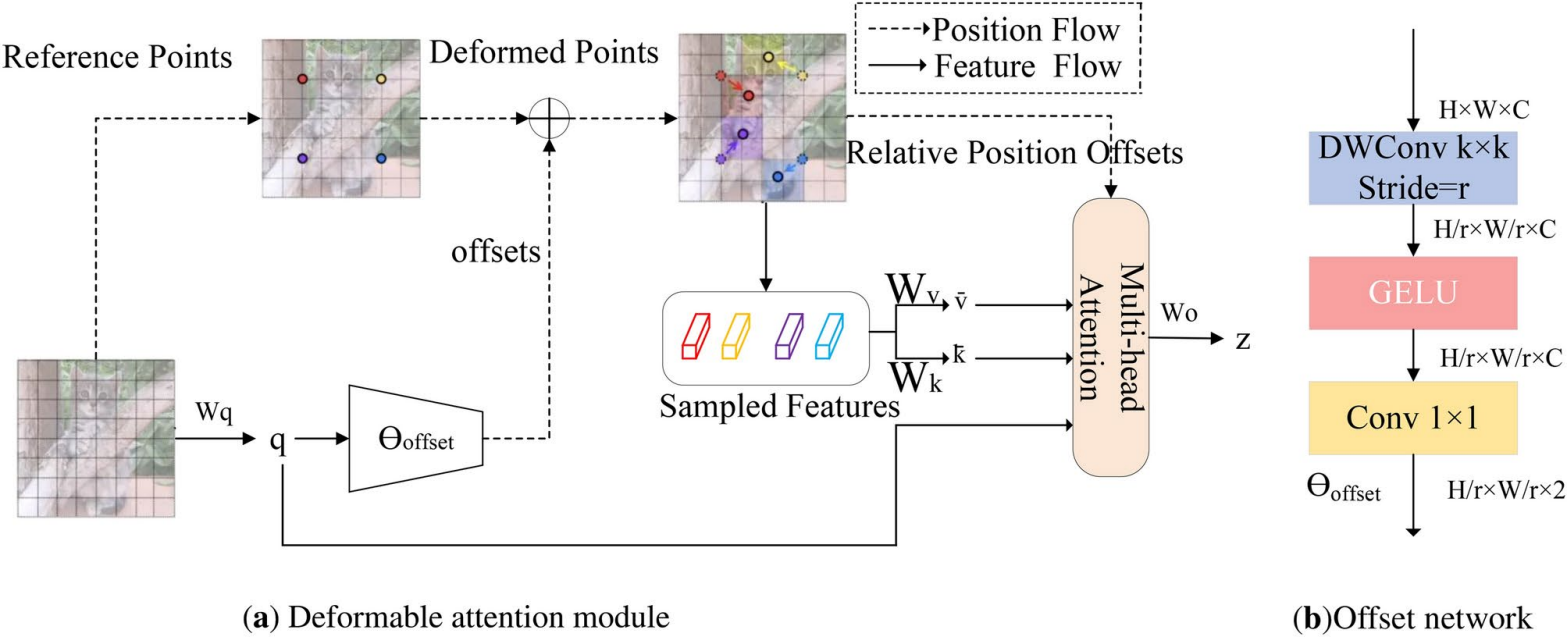
Deformable attention transformer.

The deformable attention structure is depicted in Fig. 5a, and a uniform grid of points p $\left( {H_{G} \times W_{G} \times 2} \right)$ is created as a reference given the input feature map x$\left( {H \times W \times C} \right)$. By downsampling the input feature map dimensions, $H_{G} = H/r,W_{G} = W/r$, the grid dimensions are produced. The reference point values are 2D coordinates that are linearly spaced $\left( {0,0} \right)$,...,$\left( {H_{G} - 1,W_{G} - 1} \right)$. These values are then normalized to the range [$-$1,+1] in accordance with the $H_{G}\times W_{G}$ mesh shape, where the lower right corner is represented by (+1, +1) and the top left corner by ($-$1,$-$1). To obtain the [offset] for each reference point, the input feature map is linearly projected to generate the query token, which is then processed by a lightweight sub-network ($\theta offset$) to produce the offsets. A sub-network is employed for offset generation, taking the query as input and producing the offset value for the reference point as output. Given that each reference point covers a local $s \times s$ region (where *s* is the maximum offset value), the generated network must effectively capture local features to learn appropriate offsets. The sub-network is implemented using two convolutional modules with nonlinear activation, as shown in Fig. 5b. The input features are first processed by a $k \times k$ deep convolution to capture local features, followed by the generation of 2D offsets using GELU activation and a $1 \times 1$ convolution. The amplitude of the limits is constrained by a predefined factor *s* to prevent excessive offsets, after which features are sampled at the positions of the deformed points as keys and values:

3 $$\begin{aligned} \displaystyle q= & x W_{\textrm{q}}, \quad \widetilde{k} = \widetilde{x} W_{\textrm{k}}, \quad\widetilde{v} = \widetilde{x} W_{v} \end{aligned}$$

4 $$\begin{aligned} \Delta p= & \theta _{\text {offset}}(q), \quad\widetilde{x} = \phi (x; p + \Delta p) \end{aligned}$$

where $\overline{k}$ and $\overline{v}$ denote the deformed key embedding and value embedding, respectively. The sampling function employs bilinear interpolation to ensure differentiability.

5 $$\begin{aligned} \phi \left( z; \left( p_x, p_y\right) \right) = \sum _{\left( r_x, r_y\right) } g\left( p_x, r_x\right) g\left( p_y, r_y\right) z\left[ r_y, r_x, :\right] \end{aligned}$$

where $g\left( {a,b} \right) = \max \left( {0,1 - \left| {a - b} \right| } \right)$ and $\left( {{r_x},{r_y}} \right)$ index all positions in $z \in {R^{H \times W \times C}}$. It simplifies the weighted average from ${E_q}\left( 8 \right)$ to the four sites by ensuring non-zero contributions only at the four integration points closest to $\left( {{p_x},{p_y}} \right)$. This weighted average from ${E_q}\left( 8 \right)$ to the four sites serves as the basis for the overall weighted average. Similar to existing methods, multiple attention is applied to *q* , *k* and *v* , with a relative positional offset *r* incorporated. The output of the attention head is formulated as follows:

6 $$\begin{aligned} z^{(m)} = \sigma \left( \frac{q^{(m)} \widetilde{k}^{(m) \top }}{\sqrt{d}} + \phi \left( \mathop {B}\limits ^{\wedge }; R\right) \right) \widetilde{v}^{(m)} \end{aligned}$$

Here, $\phi \left( {\hat{B};R} \right) \in {R^{HW \times H_{G}W_{G}}}$ corresponds to the positional embedding. Features from each head are adapted and connected, with the final output *z* obtained by projecting through ${W_o}$. This approach shifts the candidate key/value to important regions, enhancing the flexibility and efficiency of the original self-attentive module to capture more informative features.

Compared to traditional self-attention, deformable attention dynamically adjusts the weights in the feature mapping by adaptively focusing on the importance of each region through learnable offsets to enhance the capture of small target features. In addition, it supports multi-scale feature fusion, which can simultaneously capture the global information of a large target and the local details of a small target, thus improving the detection capability in complex scenes.

### Integration of small target detection layer

The deep network in the RTDETR model extracts abstract semantic feature information, capturing large target characteristics, while the shallow network focuses on detailed features that encompass more information about small targets. Although the deformable attention mechanism emphasizes feature information of small targets in high-resolution downhole images, this information gradually diminishes with increased downsampling, making it challenging for the model to effectively detects and classifies small objects within deeper layers of feature maps. To cope with the above problems, the original RTDETR model was designed with three different scales of detection heads, corresponding to $80\times 80$, $40\times 40$, and $20\times 20$ feature map scales, respectively. The purpose of this design is to improve the detection capability of the model for targets of different sizes by processing targets of different scales with different detection heads. However, although this strategy can solve the problem of detecting large targets to some extent, there are still challenges in detecting the accuracy of small targets.

**Fig. 6:**
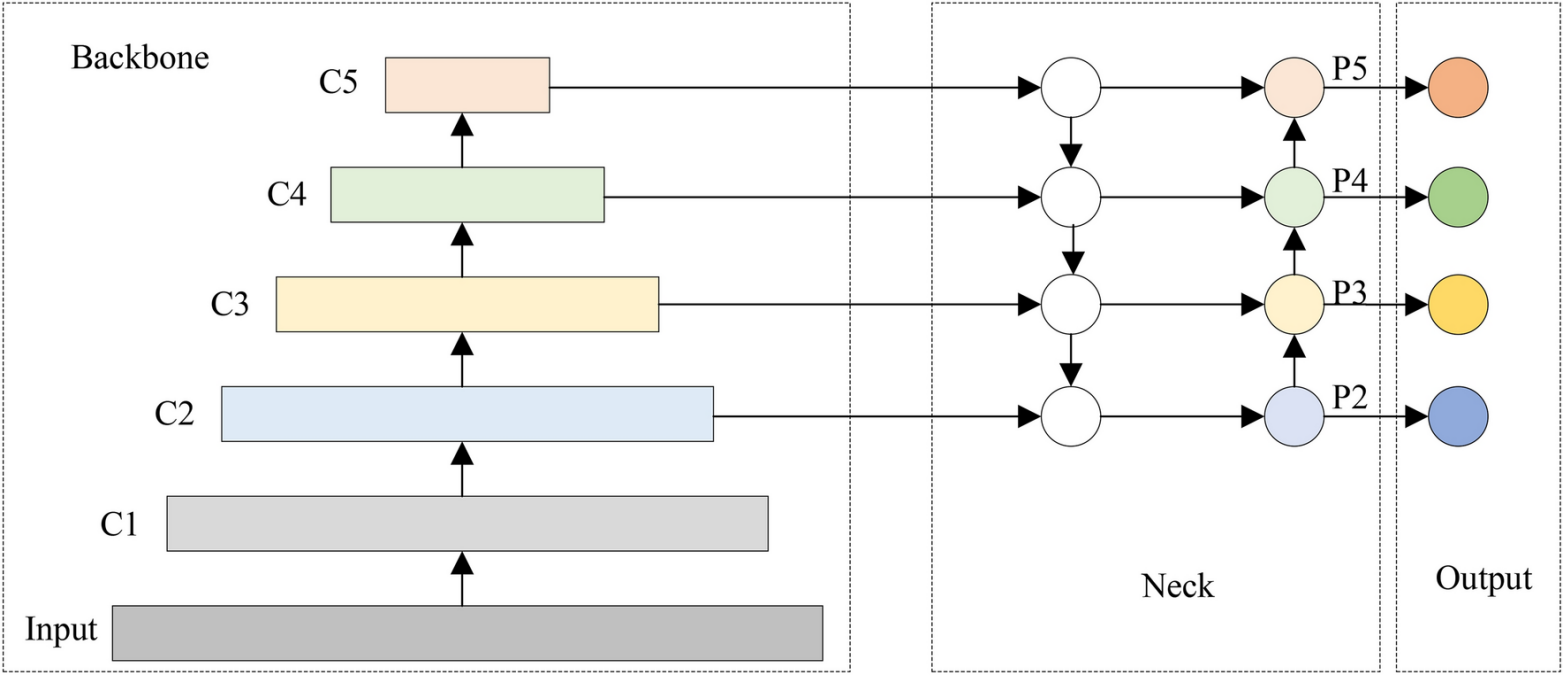
Structure of P2 detection layer in PDP-RTDETR.

To address the above problems, this paper adds a small-target detection layer to Encoder. In the efficient hybrid encoder structure of the model, P2 works together with the deformable attention-based in-scale feature interaction module to fuse the rich small-target semantic information from shallow features with the global information from the deeper semantic layer of the $160 \times 160$ scale, which is an innovative architecture that efficiently overcomes the information loss normally This innovative architecture effectively overcomes the information loss usually associated with traditional convolution and downsampling processes and significantly improves the resolution and small target detection capability of the model. The structure of the P2 detection layer in PDP-RTDETR is shown in Fig. 6.

## Experiment results and analysis

### Experimental environment configuration

The experiments were conducted on the Ubuntu 20.04 operating system, utilizing an AMD EPYC 7601 CPU and an NVIDIA RTX 3060 graphics card with 12GB of video memory. The experiment utilized the PyTorch deep learning framework, with a development environment comprising PyTorch 1.13.1, CUDA 11.7, and Python 3.8. The input image dimensions were set to 640 x 640 during training, with a batch size of 16 and 200 epochs in total. The Adam optimizer was utilized, with all other parameters set to default. The specific configuration is presented in Table 1.

**Table 1:** Experimental environment and configuration details.

Experimental environment	Configuration
GPU	NVIDIA RTX 3060 (12GB)
CPU	AMD EPYC 7601
Operating system	Ubuntu 20.04
GPU environment	CUDA 11.7
Deep learning framework	PyTorch 1.13.1
Compiler	Python 3.8
Epochs	200

### Dataset construction

**Fig. 7:**
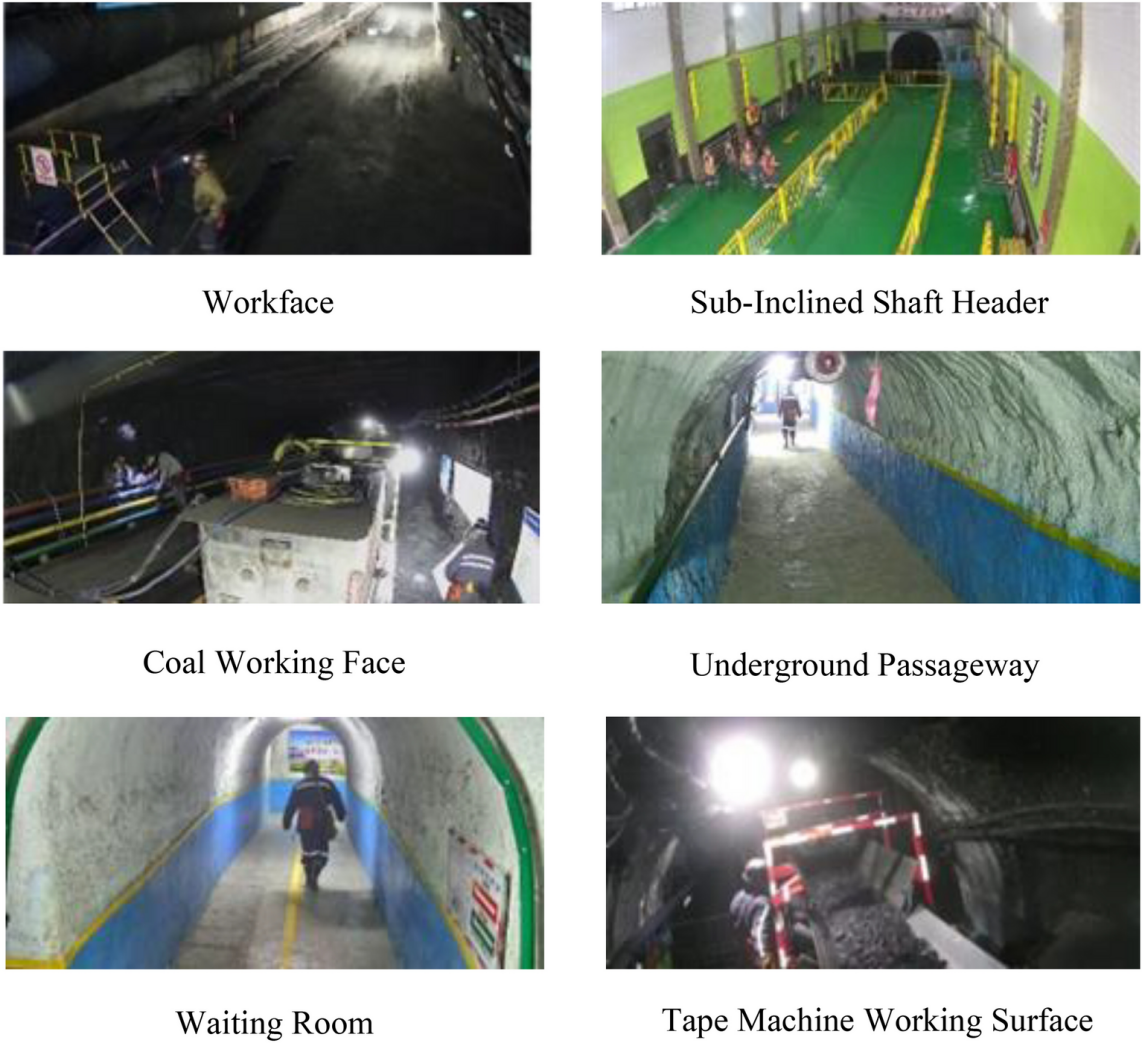
Sample Images from various scenarios.

The dataset used in this experiment is divided into two parts: CUMT-HelmeT^21^, a coal mine underground helmet dataset from the Research Centre for Intelligent Detection and Pattern Recognition of China University of Mining and Technology, and DsLMF^22^, a publicly available dataset of the anomalous state image dataset of the comprehensive mining face created by the team of Prof. Yang Wenjuan of Xi’an University of Science and Technology. The video images taken by several cameras placed in a coal mine’s mine operation area are used to create the second section. These datasets comprise video images captured by surveillance cameras in various underground coal mine scenarios, including the coal mining face, underground roadway, sub-slope shaft entrance, underground waiting room, digging face, and tape machine face, as illustrated in Fig. 7.

### Model training

**Fig. 8:**
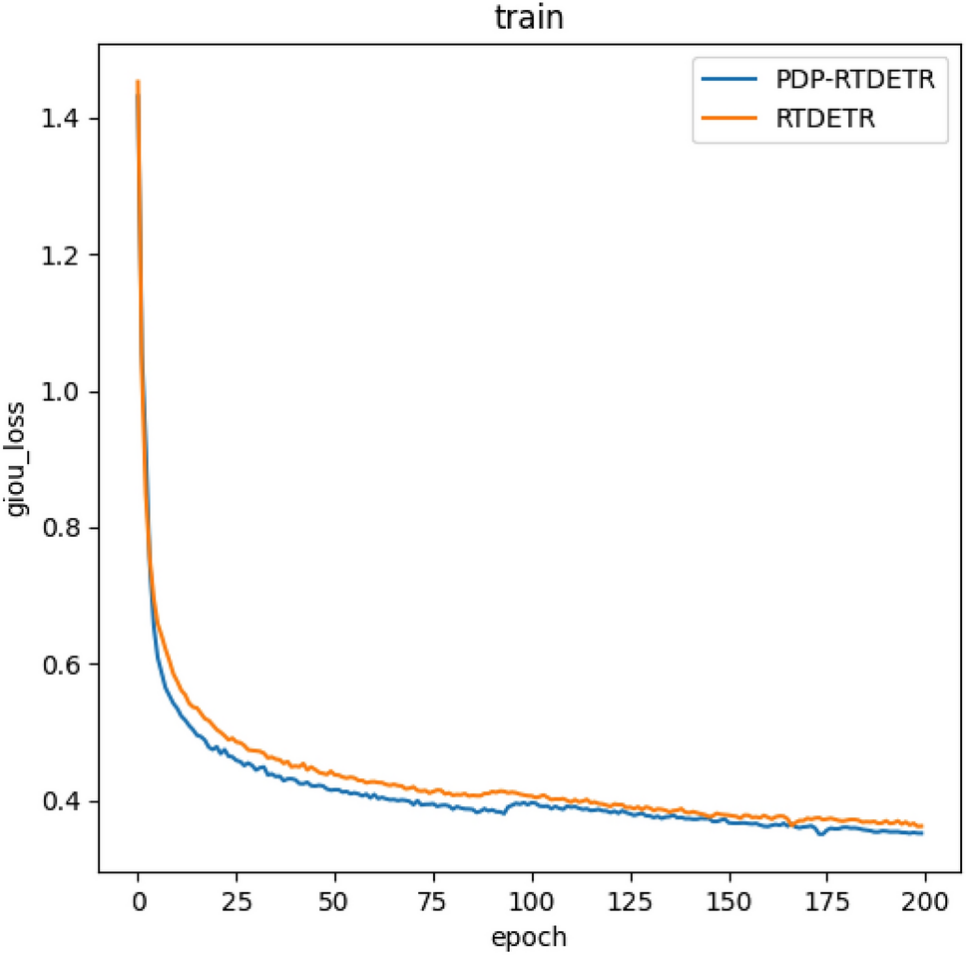
Comparison of training loss function curves.

To visualize the advantages of the PDP-RTDETR algorithm, the loss curves of both the PDP-RTDETR and the original RTDETR algorithms are presented in Fig. 8. It shows that the loss function value decreases significantly during the first 50 iterations of model training. From the 50th to the 180th iterations, the rate of decrease levels off, and in the final 20 iterations, the loss function value stabilizes. The final loss function value for the proposed model is below 0.35, while RTDETR converges near 0.40. This indicates that the training parameters for the model in this study are appropriately set, resulting in improved learning performance. The results indicate that PDP-RTDETR achieves lower loss values during training, demonstrating superior training performance.

### Evaluation indicators

To evaluate the model’s effectiveness in detecting small downhole targets, the primary indicators selected are Precision (P), Recall (R), and mAP. Additionally, model size (number of parameters), detection speed Frames Per Second (FPS), and computational complexity Giga Floating-point Operations Per Second (GFLOPs) are used as performance indicators for model inference. The mAP, P, and R serve as standard metrics for assessing target detection algorithm performance, calculated using the following formulas:

7 $$\begin{aligned} P= & \frac{N_{TP}}{N_{TP} + N_{FP}} \end{aligned}$$

8 $$\begin{aligned} R= & \frac{N_{TP}}{N_{TP} + N_{FN}} \end{aligned}$$

9 $$\begin{aligned} \text {mAP}= & \frac{\sum \nolimits _{i=1}^c AP_i}{c} \end{aligned}$$

The compiled dataset contains a total of 5600 images, divided into training, validation, and testing sets in an 8:1:1 ratio to provide adequate data for model training. The dataset was labeled using CVAT and Docker tools, with the categories including helmet and self-rescuer, focusing on small target sizes.

### Comparative experiments

**Fig. 9:**
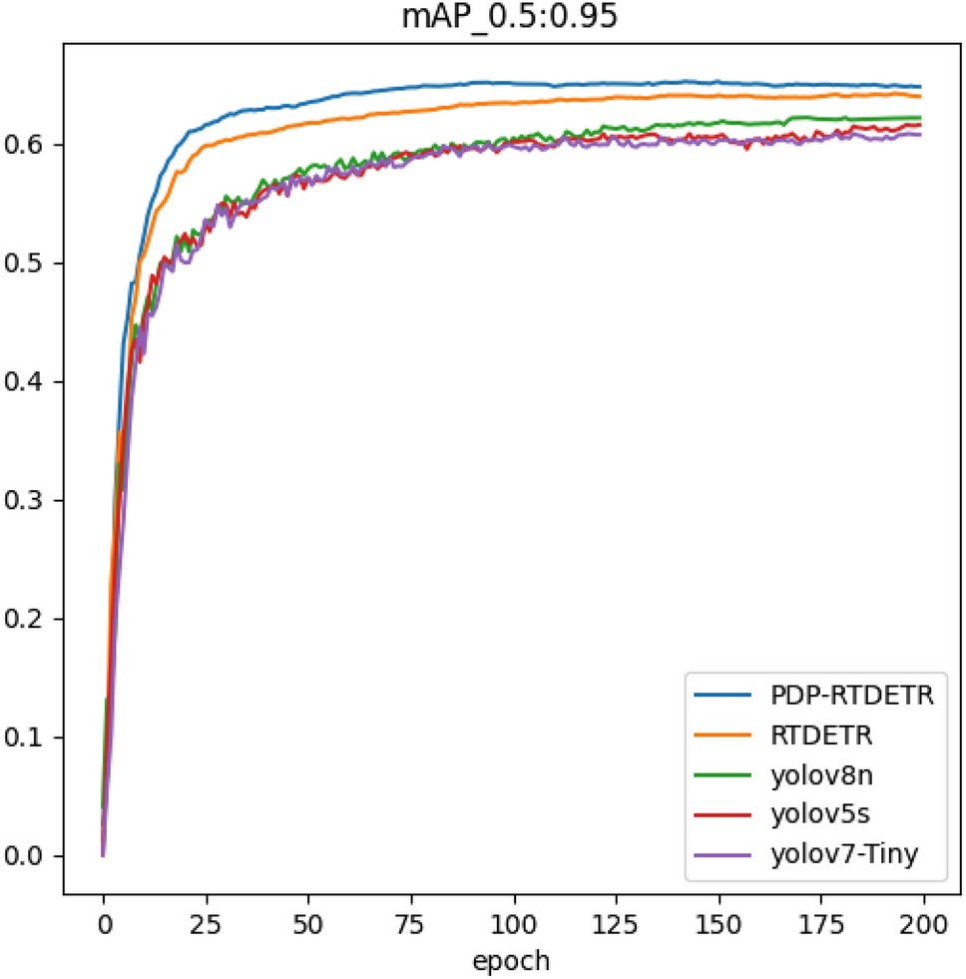
Comparison of the average accuracy of the models.

To confirm that the enhanced model that has been suggested is successful in locating tiny objects in underground mines, it is compared with advanced target detection models including Yolov5s, Yolov7-Tiny, Yolov8-n, and RTDETR. All models are trained and tested on the same dataset, with a consistent experimental environment and parameter settings. The specific experimental results are presented in Table 2. While the average accuracy mean curves of different model training are plotted as shown in Fig. 9.

**Table 2:** Comparison results of detection performance of each model.

Method	Precision %	$\hbox {mAP}_{0.5:0.95}$ %	FPS	GFLOPs	Params $10^6$
Yolov5s	88.3	45.4	87.5	70.1	25.0
Yolov7-Tiny	81.4	44.5	86.6	93.1	36.4
Yolov8n	81.8	46.7	94.0	70.2	30.0
RTDETR	90.9	51.4	31.3	64.2	19.8
PDP-RTDETR	93.8	56.6	28.2	56.9	17.2

The results in Table 2 show that compared with the mainstream algorithms, the model proposed in this paper, mAP@0.5:0.95, improves by 11.2, 12.1, 9.9, and 5.2% compared with the Yolov5s, Yolov7-Tiny, Yolov8-n algorithms, and the base model RTDETR, respectively, and the number of parameters compared to the base model decreases by 2.6M, indicating that the model proposed in this paper effectively improves the detection effect while reducing the number of parameters and performs superiorly in the task of detecting small targets under the mine. Although the detection speed of PDP-RTDETR is slightly lower than that of the base model, it still meets the real-time monitoring requirements, and the number of parameters and computational complexity are lower than those of other models, which makes it better adapted on edge devices with insufficient computational power.

### Ablation study validation

In order to verify the impact of different improvement methods on the performance of the RTDETR network model, ablation experiments were performed on the test set. The RTDETR network was used as the base model A. Models A+B (backbone), A+C (AIFI), A+D (P2), A+B+C (backbone, AIFI), A+B+D (backbone, P2), A+C+D (AIFI, P2), and A+B+C+D (backbone, AIFI, P2) were improved, and changes in the evaluation indexes of the eight models were quantified. The experimental results of the model on the test set are shown in Table 3.

**Table 3:** Results of Ablation experiments.

Modeling	Precision %	Recall %	$\hbox {mAP}_{0.5:0.95}$ %	FPS	GFLOPs	Params $10^6$
A	90.9	82.8	51.4	31.3	64.2	19.8
A+B	89.5	79.9	50.4	35.5	42.8	14.0
A+C	91.6	83.2	52.4	30.8	68.1	20.1
A+D	92.0	84.5	53.1	31.0	78.1	21.6
A+B+C	90.2	83.0	51.6	31.9	43.3	15.1
A+B+D	91.7	83.4	52.4	34.8	52.3	15.3
A+C+D	94.2	87.7	57.4	25.0	80.1	22.0
A+B+C+D	93.8	87.5	56.6	28.2	56.9	17.2

Based on the data analysed in Table 3, it can be observed that the P2 module with the deformable attention mechanism performs well in improving the accuracy of the model, while the PConv module performs well in reducing the amount of model computation. When the P2 module is used in conjunction with PConv, the model computation is reduced by 4.5M compared to the base model RTDETR. Deformable Attention is reduced by 4.7M when used in conjunction with PConv. The combination of the P2 module, Deformable Attention, and the PConv method resulted in a 2.6M reduction in model computation, along with a 5.2% improvement in mAP. In summary, the combination of the P2 module, Deformable Attention, and PConv not only reduces the amount of computation but also maintains the accuracy of the model.

### Comparison of detection performance

**Fig. 10:**
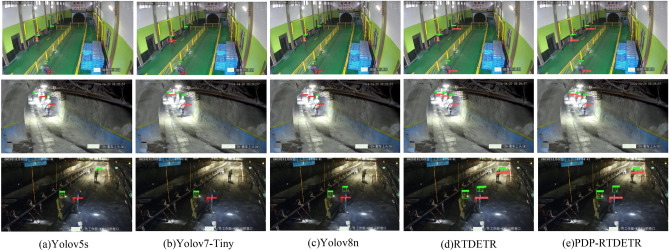
Visual comparison of model detection performance.

To intuitively illustrate the detection performance of the PDP-RTDETR model in coal mine video surveillance, visual comparisons were made between the Yolov5s, Yolov7-Tiny, Yolov8n, RTDETR, and PDP-RTDETR models, as shown in Fig. 10.

From the detection map, we can see that the detection targets in each scene account for fewer pixels in the whole image, which can easily lead to the basic algorithm producing leakage and wrong detection phenomena. The algorithm adopted in this paper introduces a small target detection layer, which is more conducive to the expression of the characteristics of the small target, and at the same time, in the algorithm, it joins the deformable attention mechanism, which makes the model pay more attention to the important information and the important position, thus effectively reducing the leakage due to external interference or dimming conditions. Reduce the leakage of detection due to external interference or dim conditions. The comparison of the three sets of images shows that the Yolov5s, Yolov7-Tiny, Yolov8n and RTDETR algorithms have different degrees of missed detection and false detection in complex environments, and the improvedPDP-RTDETR model effectively solves this problem. The PDP-RTDETR model effectively meets the requirements for downhole small target detection tasks.

### Discussion

Coal mine safety production has always been the top priority of enterprise production work, and the wearing of safety equipment is of great significance to improve the safety of mine workers. The detection of safety helmets and self-rescuers based on deep learning is an important research direction of coal mine safety production. Based on the RTDETR model, partial convolution, deformable attention mechanism and small target detection layer, this paper uses a large number of real mine data to build a self-built dataset. Finally, the improved algorithm is significantly enhanced in terms of average precision and parameter quantity, which proves the effectiveness of these modules. However, the current algorithm is certainly not the optimal solution, and there is still more room for improvement, which is worthy of further discussion.

Lightweight design, as the comparison results show, although the improved algorithm still meets the real-time standard, but compared with the original model and other models, the FPS is reduced, which is a key point that cannot be ignored, based on this problem, we consider the next step to further explore the convolution module and network structure, and consider whether more lightweight modules can be used to reduce the computational cost.

### Conclusion

In an attempt to overcome the problems with tiny objects and poor accuracy due to difficulties in feature extraction in underground coal mine safety equipment detection, this study proposes an improved algorithm based on PDP-RTDETR for detecting safety helmets and self-rescuers. The BasicBlock and PConv modules in the backbone network are fused to enhance detection speed and accuracy, making the model more suitable for small target detection. A new small target detection layer is incorporated into the encoder head, and a deformable attention mechanism is integrated into the internal feature scale interaction module. This approach facilitates better extraction of detailed information from both deep and shallow feature maps, thereby improving model detection accuracy. Subsequent investigations may augment the precision and resilience of the refined PDP-RTDETR based algorithm for small target identification in intricate situations within coal mines by means of multimodal data amalgamation, expansion of application scenarios, and improvement of computational resources and efficacy.

Future work will focus on further improving the model’s performance, and while the approach proposed in this paper has some advantages, it also has limitations. Although the PConv module effectively reduces the computational burden, it may lead to performance degradation in some cases. Therefore, in scenarios with low computational complexity, the model may sacrifice some of the accuracy and robustness of the detection. Future research hopes to further improve the model’s performance while ensuring computational efficiency.

## Data Availability

The public datasets DsLMF and CUMT-Helmet used in this study can be found at: https://www.kuangyeren.net/102593.html and https://baijiahao.baidu.com/s?id=1762471630777582587&wfr=spider&for=pc.
